# Pit Ponies

**Published:** 1911-02-18

**Authors:** Francis A. Cox

**Affiliations:** Hon. Sec. National Equine Defence League, 27 Beaconsfield Road, New Southgate, London, N.


### EDITOR'S LETTER-BOX.

### PIT PONIES.

*To the Editor of* THE HOSPITAL.

SIR,—With reference to the letter on this subject in your issue of the 21st Jan., I should be glad if you would allow me to place before your readers the proposed restrictions and regulations for effecting a reform of the present treatment and conditions of pit ponies which I tendered to the Royal Commission on Mines at my examination on the 12th, 13th and 19th July :—

1. The numbering of every animal on descending the shaft.
2. No animal to be engaged under or over a certain age.
3. Limitation of hours of ponies' work.
4. A meal midway between the allotted hours of work.
5. Supply of water for every animal engaged.
6. Every driver to be licensed and registered and the time during which he is in charge of a specified animal booked to him.
7. Every injury to a pony to be reported, investigated, and registered.
8. Periodical examination of all animals by veterinary surgeons appointed by Government, and invested with plenary powers.

These suggestions are based on the consensus of the opinions expressed by the miners who have been giving us their essential evidence and assistance in this work.

I would desire to point out that we are fully aware that some of them are at present voluntarily included in the "Special Rules" observed at various collieries, and that their legal adoption and enforcement would not in the least degree affect the collieries at which the well-being of the animals employed is considered, but would certainly preclude the perpetration of deliberate or necessitated cruelty by the remainder. The number and extent of these latter is the question which is the main consideration. But whatever facts may be elicited, our contentions are plain and decided. At the present time animals in mines owe their sole protection to these perfunctory "Special Rules" which may be suggested by the colliery management; the human workers are protected in their work by the most precise and circumspect code of regulations that can be devised by the wit of man, legally enacted, universally applied, and stringently enforced. We desire to put the claims of the dumb worker on a legislative level with those of the human, and by identical means. This duty should not be entrusted to the R.S.P.C.A., to any other society, or even to the National Equine Defence League. As a national, social, and moral obligation, its realisation should be effected by the nation, through the nation's appointed officers, and most certainly not by private charity. Societies die—or should—of their own success, and are to a very large degree dependent for their efficiency on the character and inclinations of their paid officials (whose hearts may—or may not—be in their work), who have to conform to the whims and prejudices of a committee, and who, moreover, may exploit the cause as a basis for eliciting public money. As a nation, we have no right to ask or expect a small section of the public to burden themselves with the execution of a duty which is incumbent upon us all.—Yours faithfully,

FRANCIS A. COX,

National Equine Defence League, Hon. Sec.

27 Beaconsfield Road,

New Southgate, London, N.

